# Study on the Synthesis and Properties of Waterborne Polyurea Modified by Epoxy Resin

**DOI:** 10.3390/polym14112283

**Published:** 2022-06-04

**Authors:** Jing Wang, Jihu Wang, Song Wang, Shaoguo Wen, Kaimin Chen, Chen Xie, Chunping Yuan

**Affiliations:** College of Chemistry and Chemical Engineering, Shanghai University of Engineering Science, Shanghai 201620, China; wangjingwangyi2022@163.com (J.W.); ws1816638231@163.com (S.W.); sgwen1@sues.edu.cn (S.W.); kmchen@sues.edu.cn (K.C.); xiechen03211999@163.com (C.X.); 34210005@sues.edu.cn (C.Y.)

**Keywords:** waterborne polyurea, epoxy resin, solvent resistance, mechanical properties, thermal stability

## Abstract

The most notable features of polyurea are its fast reaction, energy-saving and high efficiency. In order to meet the needs of environmental protection, waterborne polyurea (WPUA) has become a research hotspot. However, the presence of hydrophilic groups in WPUA reduces its solvent resistance, heat resistance and mechanical properties. Therefore, it is necessary and valuable to develop a high-performance WPUA. In this study, epoxy-modified waterborne polyurea (WPUAE) emulsions were prepared using epoxy resin as a modifier. Fourier transform infrared spectroscopy (FT-IR) showed that E44 was successfully introduced into the molecular chain of WPUA. The WPUAE was tested for gel fraction, adhesion, contact angle, solvent resistance, tensile properties and thermal stability. The results showed that when the E44 content was 8 wt%, the performance of WPUAE was best, the adhesion of WPUAE coating film was 1.53 MPa, the gel fraction, water contact angle, water absorption, toluene absorption, tensile strength and decomposition temperature were 96.94%, 70.3°, 16.43%, 131.04%, 9.05 MPa and 365 °C, respectively. The results showed that epoxy resin as an emulsion modifier improved the comprehensive properties of WPUA.

## 1. Introduction

Polyureas are known to be made from isocyanates and polyamines [1]. However, the group of -NCO reactions with amine is very rapid, and special equipment is needed for spraying polyureas. In order to solve this problem, the dispersion of polyurea in water to obtain waterborne polyurea (WPUA) has become a hot research topic. WPUA is used in many fields as an environmentally friendly material. As we all know, WPUA contains the macromolecular chain structure with hydrophilic groups, resulting in the poor solvent resistance, mechanical properties and thermal stability of WPUA films, which would limit their application [2,3]. So many methods are used to modify WPUA [4,5,6]. Kalmagambetova et al. [7] studied the polyurea modified by hollow glass beads, which improved the thermal insulation, corrosion resistance and water resistance of the polyurea coatings effectively. Zhao et al. [8] reported using polydimethylsiloxane, inert silicone oil and 1,3,5-tris-(4-aminophenoxy) benzene as a modifier, lubricant and crosslinking agent, respectively, to prepare polyurea. The polyurea coating has the properties of low ice shear strength, high mechanical stability and high self-healing. Zhang et al. [9] synthesized the waterborne polyurea modified by sulfonated graphene with the network barrier structure, which effectively improved the corrosion resistance of the coating films. Arzakov et al. [10] reported polyurea modified with polytetrafluoroethylene powder and polydimethylsiloxane. The modulus of elasticity can be controlled from 22 MPa to 172 MPa, which made WPUA turn from rubber to plastic.

Epoxy resin has the advantages of solid adhesion, dense molecular structure, good stability, low shrinkage, high modulus, high strength and so on [11,12]. It is widely used in coatings, engineering plastics and adhesives [13,14]. In addition, there are many successful applications of epoxy resins as modifiers. Li et al. [15] synthesized waterborne epoxy modified bitumen emulsion (WEB) and analyzed the creep and recovery behavior of the WEB residues under high-temperature conditions. The results showed that the high-temperature performance of WEB residues was improved significantly. Liu et al. [16] investigated epoxy modified waterborne polyurethane (EPU) and found that the thermal stability, flexibility and storage stability of EPU were better than unmodified polyurethane. EPU as a carbon fiber sizing agent improved the mechanical interfacial strength of the matrix, changed the crack expansion path and prevented the diffusion of excessive stresses in the cracks effectively. Wu et al. [17] reported bisphenol epoxy resin modified condensed poly-nuclear aromatic (COPNA) resin. The hydroxyl groups of COPNA resin reacted with the epoxy groups to increase the crosslinking density, so the heat resistance and adhesion of COPNA resin were enhanced. Therefore, epoxy resins are usually chosen as useful modifiers to improve the combined performance of the polymer.

It is an effective method to improve the overall performance of materials by polymer modification to form a crosslinked network [18]. Ring-opening reactions of epoxy groups in epoxy resins and reactions between hydroxyl groups (-OH) and isocyanate functional groups (-NCO). Therefore, in theory, epoxy resins can be successfully introduced into the WPUA molecular chain to form WPUAE with a densely crosslinked network structure and achieve the purpose of improving material properties.

As far as we know, there is little literature focused on epoxy resin as a modifier to react with WPUA. In this work, a series of WPUAE emulsions were prepared using isophorone diisocyanate (IPDI), polyetheramine (D2000), epoxy resin (E44), 2,2-dihydroxymethylbutyric acid (DMBA) and triethylamine (TEA) as the main raw materials. The properties of WPUAE were characterized by various analytical techniques, for example, the mechanical stability of the emulsions was investigated by centrifugal test, the crosslinking degree of the emulsions was characterized by gel fraction, the adhesion of coating films was evaluated by drawing adhesion test, the hydrophilic (hydrophobic) properties of the solid films were characterized by the contact angle, the water absorption and toluene absorption were used to characterize the solvent resistance of the solid films, the tensile test was used to characterize the mechanical properties of the solid films and the thermal decomposition temperature was used to characterize the thermal stability of the solid films.

## 2. Materials and Methods

### 2.1. Materials and Agents

Epoxy resin (E44) was obtained from Jiangsu Sanmu Group Co., Ltd. (Yixing, Jiangsu, China). Isophorone diisocyanate (IPDI), polyetheramine (D2000), triethylamine (TEA) and 2,2-Bis(hydroxymethyl)butyric acid (DMBA) were purchased from Aladdin Reagent Co., Ltd. (Shanghai, China). Acetone, toluene and ethylene glycol were bought from Shanghai Taitan Technology Co., Ltd. (Shanghai, China). The deionized water was used in the whole experiment. Epoxy resin was industrial grade, and all other reagents were analytical grade and used as received.

### 2.2. Preparation of Emulsions

**Figure 1:**
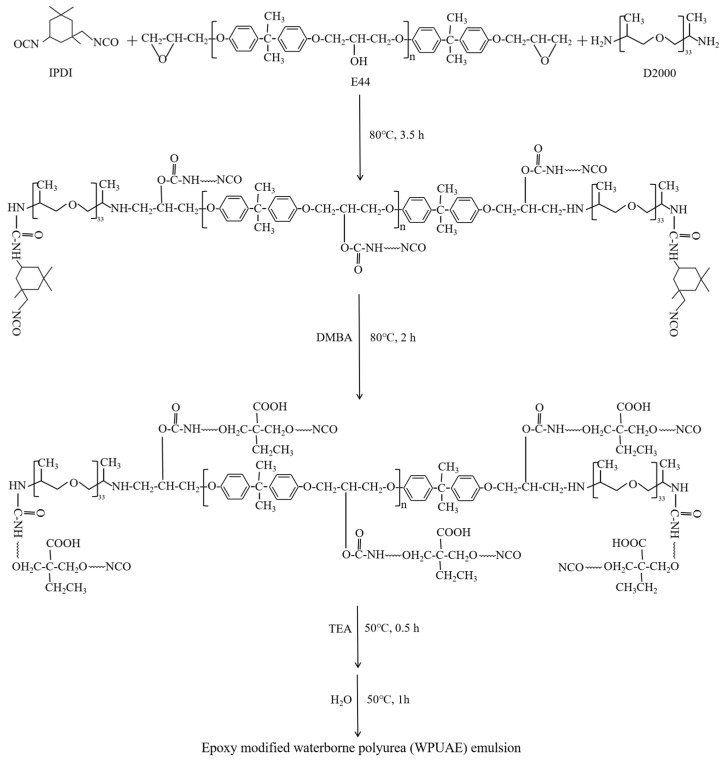
Preparation of epoxy modified waterborne polyurea (WPUAE) emulsion.

The preparation of WPUA and WPUAE emulsions followed the process shown in Figure 1. The basic experimental formulations are shown in Table 1.

Acetone was added to reduce the viscosity of the prepolymer in the whole process. Firstly, IPDI, D2000 and E44 (diluted with acetone) were added to a 250 mL three-necked flask equipped with a stirring bar and condenser. The reaction was carried out at 80 °C for 3.5 h. The content of -NCO was determined by the dibutylamine titration method [19]. When the content of -NCO reached the theoretical value, DMBA was added, and the reaction mixture continued to emulsify at 80 °C for 2 h. Then, the reaction flask was cooled to 50 °C, and TEA was added to neutralize for 0.5 h. At last, an appropriate amount of deionized water was added and dispersed under high-speed stirring for one hour to obtain the WPUAE emulsion. WPUA emulsion was prepared using the same synthesis process without E44.

### 2.3. Preparation of Films

The emulsion was applied uniformly to the polished tinplate and dried at room temperature for seven days to obtain the coating film. The thickness of the dry coating films was about 40 μm.

The emulsion was poured into the PTFE mold, which was dried at 50 °C until it reached a constant weight and cooled at room temperature to obtain the solid film. The resulting thickness was about 0.80 mm.

### 2.4. Characterization

The solid films were characterized by the ATR method in the wavenumber range of 4000~500 cm^−1^ by a Fourier transform infrared spectrometer (FT-IR, Nicolet iS5, Thermo, waltham, CA, USA). The 32 scans for each sample were obtained with a resolution of 4 cm^−1^.

The particle size and its distribution of emulsions were tested by dynamic light scattering (DLS, Zetasizer Nano, Malvern Instruments Limited, Malvern, UK).

The stormer viscosity of the emulsions was measured by a viscometer (STM-IV(B), Tianjin Huayi Xinda Instrument Co., Ltd., Tianjin, China) at room temperature.

The emulsions were centrifuged for 15 min at a speed of 3000 rpm using a centrifuge (TG18G, Shanghai Hetian Scientific Instrument Co., Ltd., Shanghai, China) to check the stability.

The emulsions were placed in a clean container and dried to constant weight, and the solid content was calculated according to the following Formula (1):

(1) $$W=\frac{m_{2}}{m_{1}}\;\times \;100\%$$

where m_1_ and m_2_ were the total mass of the emulsion before and after drying.

The adhesion of coating films was tested by a digital pull-off adhesion tester (BGD 500, Guangzhou Biaogeda Precision Instrument Co., Ltd., Guangzhou, China).

The solid films were cut into the 10 mm × 10 mm and immersed in acetone solvent for 24 h, taken out and dried to constant weight at 50 °C. The gel fraction of WPUAE was calculated by Formula (2):

(2) $$M=\frac{M_{1}}{M_{0}}\;\times \;100\%$$

where M_0_ and M_1_ were the solid film quality before and after soaking in acetone.

The water (or ethylene glycol) contact angles on the surface of solid films were measured by a contact angle meter (CA, 190-F2, Rame-Hart, Succasunna, NJ, USA). The contact angle was determined by the average value of three tests at different positions on each sample. The surface energy of solid films was calculated by the Formulas (3) and (4):

(3) $$\gamma_{S}=\gamma_{S}^{d}+\gamma_{S}^{P}$$

(4) $$\gamma_{L}\left(1+\cos\theta \right)=4\left[\left(\gamma_{S}^{d}\gamma_{L}^{d}/\gamma_{S}^{d}+\gamma_{L}^{d}\right)+\left(\gamma_{S}^{P}\gamma_{S}^{P}/\gamma_{S}^{P}+\gamma_{L}^{P}\right)\right]$$

where $\gamma_{S},\;\gamma_{S}^{d}\;$ and $\gamma_{S}^{P}$ represent the surface tension, dispersion force and polar force of the solid films, respectively. $\gamma_{L},\;\gamma_{L}^{d}\;$ and $\gamma_{L}^{P}\;$ represent the surface tension, dispersion force and polar force of test liquids, respectively. θ represents contact angle.

The dried solid films were immersed in water and toluene, respectively. They were taken out every two hours, and the residual solvent on the surface was wiped using a filter paper and then weighted immediately. Solvent absorption was calculated using Formula (5):

(5) $$S=\frac{m_{t}-m_{0}}{m_{0}}\;\times \;100\%$$

where m_0_ represents the original weight, m_t_ represents the weight after soaking.

The tensile strength and elongation at the break of the solid films were examined on a tensile testing machine (HY-025CS, Shanghai Hengyu Instrument Co., Ltd., Shanghai, China). The tensile speed was 50 mm/min. All of the test samples were prepared in a standard dumbbell shape. The results were obtained by taking the average value of three measurements.

The thermal stability of the solid films was evaluated by the thermogravimetric analyzer (TG, TGA 550, TA Instruments Co., New Castle, DE, USA) from 30 °C to 650 °C in the N_2_ atmosphere at a heating rate of 20 °C/min. The mass of each sample was 5 mg ± 1%.

## 3. Results and Discussion

### 3.1. FT-IR Analysis

**Figure 2:**
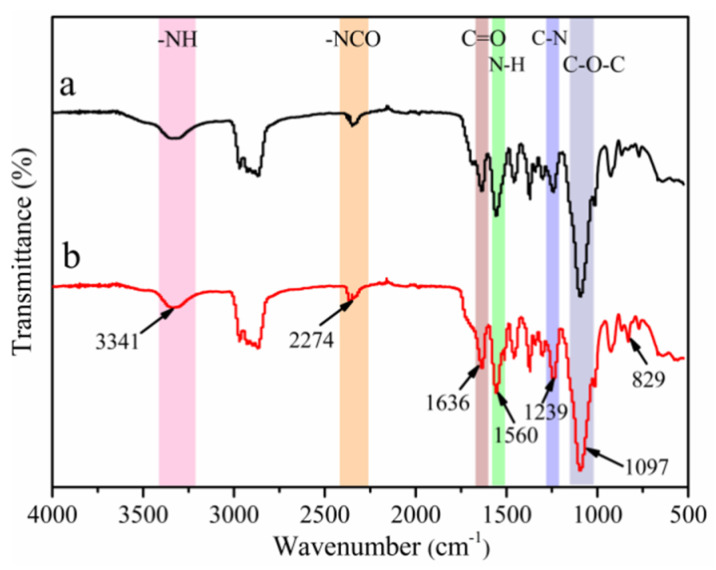
FT-IR spectra of (**a**) WPUA and (**b**) WPUAE solid films.

Figure 2 show the FT-IR spectra of (a) WPUA and (b) WPUAE solid films. The peak at 3341 cm^−1^ corresponded to the characteristic absorption peak of the -NH group. The distinct absorption peak near 2274 cm^−1^ was attributed to the stretching vibration of -NCO, indicating the presence of free -NCO in the system. The peaks at 1636 cm^−1^ (C=O stretching vibration), 1560 cm^−1^ (N-H bending vibration) and 1239 cm^−1^ (C-N stretching vibration) confirmed the formation of urea bonds [20]. The absorption peak located at 1097 cm^−1^ corresponded to the C-O-C group. The appearance of these characteristic peaks indicated that the WPUA emulsion was successfully prepared.

In Figure 2, compared with curve a, the absorption peak at 829 cm^−1^ in curve b was attributed to the bending vibration generated by the replacement of two pairs of hydrogen atoms of the benzene ring in the epoxy resin [21]. Due to the ring-opening reaction of the epoxy group, no absorption peak of the epoxy group was observed at 910 cm^−1^ [22,23]. Therefore, WPUA was successfully modified by epoxy resin.

### 3.2. Basic Performances of WPUAE Emulsions

The essential performances of WPUAE emulsions with different E44 contents are exhibited in Table 2. With the increase in E44 content, the average particle size of WPUAE emulsions increased from 204 nm to 669 nm. The viscosity was changed from 47.1 KU to 102.6 KU. However, the solid content increased from 51% to 57% when the E44 content increased to 6 wt%, then reduced to 51% when the E44 content was more than 6 wt%. The emulsions with E44 content less than 10 wt% were stable, and precipitation was observed at 10 wt%.

With the E44 content increased, more epoxy molecules were reacted with -NCO. The grafting degree and crosslinking density of WPUA were increased [24,25]. The essential performances of WPUAE emulsions were improved. However, the content of hydrophilic groups in the system remained unchanged, unreacted E44 gradually accumulated with small molecules to form agglomerates, and the particle size and viscosity of the emulsions were increased [26]. High viscosity made the phase inversion emulsification delay, so the solid content was decreased. Meanwhile, the crosslinking and phase behavior between WPUA and E44 played an important role in the stability of the emulsions, so the emulsion appeared stratified accordingly.

### 3.3. Gel Fraction Analysis

**Figure 3:**
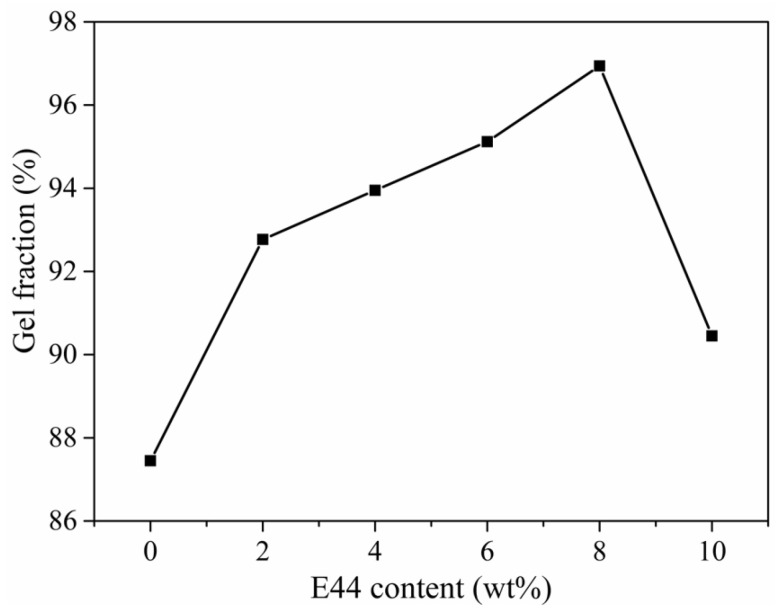
The gel fraction of WPUAE solid films.

The gel fraction test results of WPUAE solid films with different E44 contents are shown in Figure 3. The gel content of WPUAE solid films reflected the crosslinking degree of the solid films. The higher the gel fraction, the higher the crosslinking. It can be seen that the gel fraction of WPUAE solid films increased from 87.45% to 90.45% as the E44 content increased. The maximum gel fraction of solid films was 96.94% at 8 wt% E44 content. This was because that epoxy groups and hydroxyl groups in epoxy resins reacted with isocyanate functional groups (-NCO). As the content of E44 in the reaction increased, the crosslinking density of the solid films increased. The solvent molecules were difficult to enter into the crosslinking network, so the gel ratio increased.

### 3.4. Adhesion Analysis

**Figure 4:**
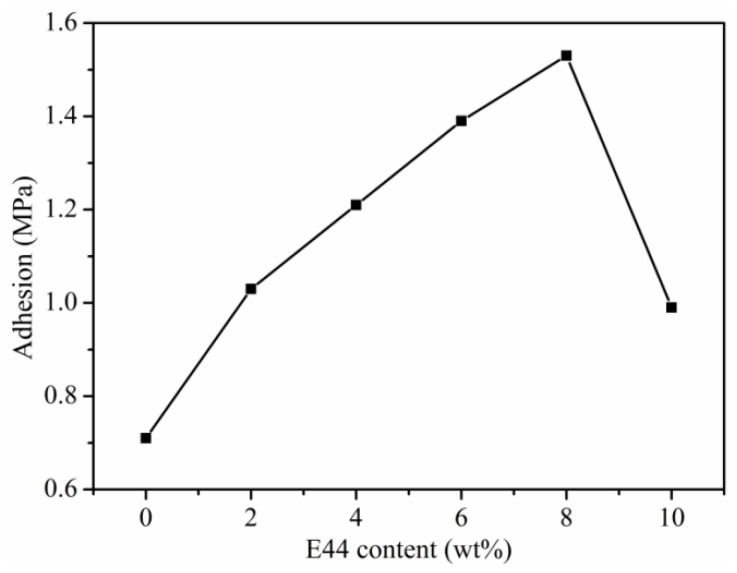
Adhesion of WPUAE coating films.

The adhesion of the coating films is vital when it is used to protect materials. The internal coating must have sufficient adhesion with the substrate to prevent moisture penetration into the coating films [27]. As shown in Figure 4, the adhesion of the WPUAE coating films increased from 0.71 MPa to 1.53 MPa as E44 increased from 0 wt% to 8 wt%. The reason is that the epoxy resin had good adhesion to the substrate [28]. The introduction of E44 can increase the crosslinking density of the emulsions and form a strong bond between the coating and tinplate [29,30]. In addition, the molecular weight of WPUAE increased with the E44 content addition. Higher molecular weights lead to better adhesion strength [31]. When the E44 content reached 10 wt%, the crosslinking degree arrived at saturation, and the structure of WPUAE became loose because of unreacted hydroxyl groups. The coating films became brittle due to the excess of the benzene ring. Therefore, the adhesion of the coating films decreased.

### 3.5. Contact Angle Analysis

**Figure 5:**
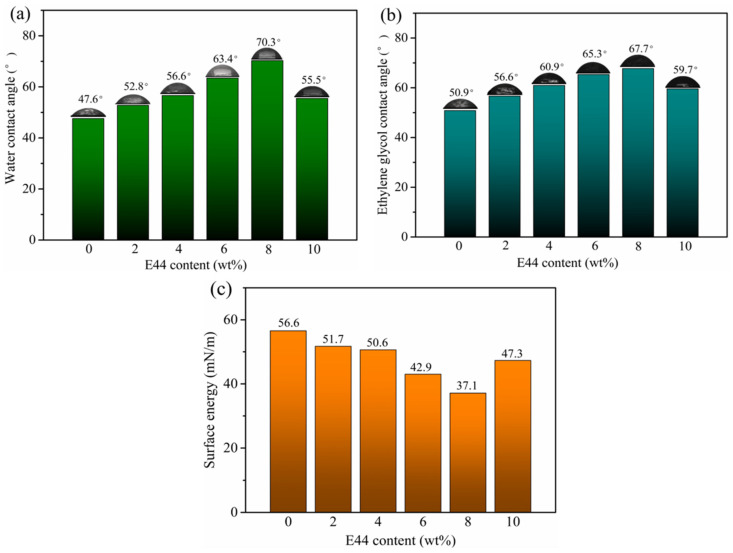
Water contact angle (**a**), ethylene glycol contact angle (**b**) and surface energy (**c**) of WPUAE solid films.

The water (ethylene glycol) contact angle and surface energy of WPUAE solid films with different E44 contents are shown in Figure 5.

With the increase of E44 content, the water (ethylene glycol) contact angles of the solid films tended to increase and then decrease. At the same time, the surface energy grew to decrease and then rose, and the maximum contact angles were 70.3° and 67.7°, respectively. The minimum surface energy was 37.1 mN/m. Because of the rigid benzene ring structure in E44, the crosslinking network structure was further enhanced as the E44 content increased, so the wettability of the solvent to the surface of the solid films was gradually reduced. When the content of E44 was excessive, the unreacted -OH in WPUAE can easy to absorb water because of the hydrogen bond. Therefore, the contact angles were reduced, and the surface free energy was increased.

### 3.6. Solvent Resistance Analysis

**Figure 6:**
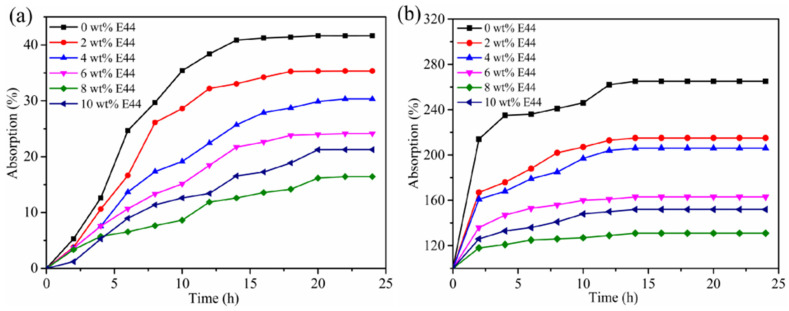
Solvent resistance of WPUAE solid films (**a**) deionized water (**b**) toluene.

The solvent resistance of the solid films was assessed by deionized water and toluene. The water and toluene absorption of the solid films are shown in Figure 6. It was found that the absorption rate of toluene was much higher than that of deionized water. The absorption rate of the solid films increased with prolonged immersion time and decreased with added E44 content. Because the addition of epoxy resin increased the molecular weight of the WPUAE chains, the crosslinking reaction and chain entanglement between WPUA and E44 led to a more compact film structure. So deionized water and toluene became difficult to transport through the solid films [32]. However, when the E44 content was up to 10 wt%, the absorption rate was slightly increased. Epoxy resin E44 contained the rigid benzene ring; therefore, much more epoxy resin could not react with isocyanate and agglomerated together to form larger clusters. It destroyed the compact degrees of WPUAE. The intermolecular gaps became larger. Another reason was that the contact angles were reduced. Therefore, the solvent could easily penetrate the surface of the solid films, resulting in decreased solvent resistance.

### 3.7. Mechanical Properties

**Figure 7:**
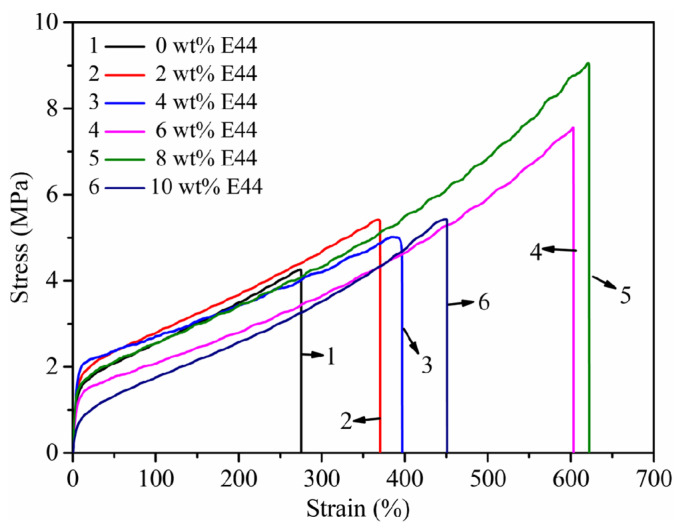
Mechanical properties of WPUAE solid films.

The tensile strength and elongation at break reflected the strengthening and toughening influence of E44 [33]. The tensile stress-strain curves of WPUAE solid films with different E44 contents are shown in Figure 7. With the increase of E44 content, the tensile strength of WPUAE increased from 4.26 MPa to 9.05 MPa, and the elongation at break increased from 275% to 622%. The reason is that the hydrogen bond and polarity of WPUAE increased as the E44 content increased. The rigidity, intermolecular force and cohesive energy of the WPUAE molecular chain were improved [34]. Furthermore, the grafting of epoxy groups into the WPUAE molecular chains by ring-opening reactions can increase the toughness of the solid films. Therefore, the tensile strength and elongation at the break of the solid films increased. When the content of E44 was 10 wt%, the emulsion system became unstable. Some of the E44 may be released from the emulsion to the water, resulting in progressively poorer compatibility of E44 with WPUA [35]. In addition, a higher proportion of hard and flexible segments were entangled, which limited the mutual sliding between the segments. Therefore, the tensile strength and elongation at the break of the solid films decreased.

### 3.8. Thermal Stability

**Figure 8:**
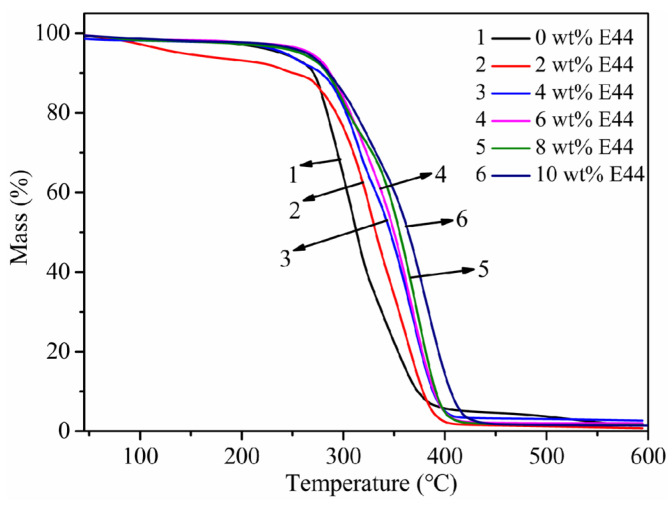
TGA curves of WPUAE solid films.

The decomposition temperatures were tested to analyze the thermal stability of WPUAE. The TGA curves of the WPUAE are drawn in Figure 8. The degradation temperatures at 10% weight loss (T_10%_), 50% weight loss (T_50%_) and 95% weight loss (T_95%_) are listed in Table 3.

As shown in Figure 8, the weight loss of WPUAE solid films at different amounts of E44 was less than 10 wt% at temperatures below 250 °C, which may be due to the evaporation of residual trace moisture and organic solvents in the sample. When the temperature was higher than 250 °C, the weight loss rate of WPUA solid films increased significantly. The thermal decomposition temperature of the WPUA solid films gradually increased as the content of E44 increased.

Compared with WPUAE and WPUA, it can be found that T_10%_ increased from 251 °C to 291 °C, T_50%_ increased from 312 °C to 365 °C and T_95%_ increased from 313 °C to 373 °C after increasing the E44 content to 10 wt% from Table 3. The thermal stability of WPUAE solid films was better than that of WPUA. The heatproof nature of WPUAE was enhanced with increased E44 content. The decomposition temperature of the polymer was related to the heat resistance of the various functional groups in the macromolecular structure. After E44 was introduced into the WPUA structure, the rigid benzene ring was increased, and the crosslinking network structure became denser. Therefore, the thermal decomposition of the molecular chains was restricted, and the thermal stability was improved [36,37].

## 4. Conclusions

In summary, WPUAE emulsions with different E44 contents were successfully prepared. After performance tests, it was found that the addition of the appropriate amount of E44 could effectively improve the comprehensive performance of WPUA, and when the addition of E44 was 8 wt%, the gel fraction was reduced from 92.45% to 79.38%, at which point the WPUA showed the best performance. WPUAE emulsions showed high stability in centrifugation tests. The adhesion of WPUAE coating films increased from 0.71 MPa to 1.53 MPa. The water absorption of WPUAE solid films decreased from 21.26% to 16.43%, and the toluene absorption decreased from 265% to 131%. The water contact angle increased from 47.6° to 70.3°, the tensile strength increased from 4.25 MPa to 9.05 MPa, the elongation at break increased from 274% to 622% and the thermal decomposition temperature increased gradually with the increase of E44 content. Therefore, the epoxy resin played a positive role in improving the overall performance of WPUA. This study may arouse interest in using epoxy resin as a modifier to improve the overall performance of waterborne emulsions.

## Figures and Tables

**Table 1:** Basic experimental formulations for the synthesis of WPUAE emulsions.

E44 Content (wt%)	Raw Material (g)				
	IPDI	D2000	E44	DMBA	TEA
0	7.00	12.50	0.00	0.78	0.60
2	7.00	12.50	0.41	0.78	0.60
4	7.00	12.50	0.82	0.78	0.60
6	7.00	12.50	1.25	0.78	0.60
8	7.00	12.50	1.70	0.78	0.60
10	7.00	12.50	2.18	0.78	0.60

Note: E44 (wt%) = [m_E44_/(m_IPDI_ + m_D2000_ + m_E44_)], “m” stands for mass.

**Table 2:** Basic performances of WPUAE emulsions.

E44 Content (wt%)	Average Particle Size (nm)	Viscosity (KU)	Solid Content (%)	Storage Stability
0	204	47.1	51	no precipitation
2	259	61.8	54	no precipitation
4	328	68.7	55	no precipitation
6	416	87.7	57	no precipitation
8	417	90.9	56	no precipitation
10	669	102.6	51	precipitation

**Table 3:** Decomposition temperature of WPUAE solid films.

E44/wt%	0	2	4	6	8	10
T_10%_/°C	251	271	278	288	284	291
T_50%_/°C	312	332	334	350	355	365
T_95%_/°C	313	361	362	365	365	373

## Data Availability

The data presented in this study are available in the article.
